# The effect of frailty on the 24‐hour blood pressure pattern in the very elderly

**DOI:** 10.1111/jch.14409

**Published:** 2021-12-09

**Authors:** Fernando Gioppo Blauth, Laís Araújo dos Santos Vilar, Victor de Carvalho Brito Pontes, Júlio César Moriguti, Eduardo Ferriolli, Nereida Kilza da Costa Lima

**Affiliations:** ^1^ Division of Internal Medicine and Geriatrics Department of Internal Medicine Ribeirão Preto Medical School University of São Paulo, Ribeirão Preto Brazil

**Keywords:** aged 80 and over, ambulatory blood pressure, blood pressure dipping, frailty, hypertension in the elderly

## Abstract

Frailty plays a crucial role in the management of hypertension in the very elderly and has a strong association with cardiovascular diseases. Nevertheless, its influence on the 24‐hour blood pressure pattern, including elevated asleep systolic blood pressure (BP) and the lack of BP fall during sleep (non‐dipping) has not been explored in a population above 80 years.

Patients older than 80 years were classified into frail or robust subtypes by the five item frailty phenotype criteria. All participants were submitted to office blood pressure measurements and ambulatory BP monitoring over a 24‐hour period. Nocturnal dipping was defined as nighttime BP fall ≥10%.

Thirty‐eight frail and 36 non‐frail individuals (mean age 85.3 ± 3.7 years; 67% females) were analyzed. Awake systolic and diastolic BP were similar for frail and robust individuals. Frail patients had higher systolic BP during sleep (128 ± 15 mm Hg vs. 122 ±13 mm Hg *p* = .04) and reduced systolic BP fall [1 (‐4.5 – 5)% vs. 6.8 (2.1 – 12.8)% *p* < .01]. Frailty was independently associated with higher risk of non‐dipping (OR 12.4; CI 1.79 – 85.9) and reduced nighttime systolic BP fall (‐6.1%; CI ‐9.6 – ‐2.6%). In conclusions, frailty has a substantial influence on nighttime BP values and pattern in patients older than 80 years.

## 1 INTRODUCTION

Despite significant advances in treating cardiovascular diseases (CVD), hypertension continues to be a burdensome disorder, leading to substantial loss of independence and representing a great onus for health systems worldwide.^1^, ^2^, ^3^ With the emergence of population ageing, this fact has become more eminent. There is a direct relation between age and hypertension, with some studies showing a prevalence of approximately 82% in individuals above 75 years.^3^, ^4^

Although there is evidence for treating individuals older than 80 years, the paucity of clinical trials in the very elderly, the high prevalence of polypharmacy and cognitive disorders and the wide variability amongst individuals represent a challenge in managing hypertension in this specific population.^5^, ^6^, ^7^ Frailty scores, as instruments used to assess biological age, play a crucial role in interpreting and managing health problems in this population.^8^, ^9^, ^10^

Studies show that well known CVD behave differently between the frail and robust population regardless of age. While the frail population tends to have higher cardiovascular risk, greater disease burden and increased disability and mortality; the non‐frail of the same age behave in an opposite manner.^11^, ^12^ Many theories have raised plausible explanations for the association between frailty and CVD but few studies have used clinical measures along the circadian cycle such as 24‐hour ambulatory blood pressure monitoring (ABPM) in order to obtain a better understanding of the problem.^10^

Elevated asleep systolic blood pressure (BP) and the lack of BP fall (dipping) have been associated with poorer cardiovascular outcome and are, amongst ABPM findings, the best prognostic markers of future cardiovascular events.^13^, ^14^, ^15^, ^16^ Studies have indicated that older patients have higher nighttime systolic BP as well as a reduced dipping and we hypothesize that these findings might be influenced by frailty.^8^, ^17^

No studies have been designed so far to analyze the role of frailty on these important cardiovascular risk markers in individuals above 80 years old. Therefore, the objective of the present study was to examine the effect of frailty on the 24‐hour blood pressure profile, in a population above 80 years, with specific focus on nighttime BP values and pattern.

## 2 METHODS

### 2.1 Participants

All participants were enrolled from March 2019 to April 2021 at a geriatric clinic of the University Hospital, University of São Paulo, located in the city of Ribeirão Preto, São Paulo, Brazil. Participants included in the study were any patient older than 80 years classified into frail or robust subtype. Exclusion criteria were based on circumstances that could interfere with the 24‐hour blood pressure measurement or with the natural sleep pattern such as atrial fibrillation, being bedridden, inability to walk, advanced cognitive disorders, presence of obstructive sleep apnea or use of breathing devices for sleep.

Data collection was suspended during local restrictions due to COVID‐19 pandemic outbreak. When the study was resumed, proper safety measures were taken, with special care that all included individuals were asymptomatic at the time of the analysis.

The study was approved by an independent Ethics Committee (protocol no. 3.500.611) and all patients gave written informed consent to participate in the study. A total of 120 individuals were first assessed for frailty and 77 of them met the inclusion criteria and were invited to participate in the study. Three had to be excluded due to insufficient ABPM measurements, with the final sample thus consisting of 74 patients.

All participants were submitted to comprehensive evaluation including comorbidities, medications in use, weight, height, abdominal circumference, as well as office blood pressure measurements in the sitting, lying, and standing position according to current guidelines.^1^ Orthostatic hypotension was assessed with successive blood pressure readings at the first and third minute after standing. Subsequently, ambulatory blood‐pressure monitoring was performed. Comorbidities were classified into 17 categories according to the modified Charlson Comorbidities Index without weighting, in a way the variable ranges from 0 to 17.^18^

### 2.2 Frailty assessment

Frailty was assessed by the phenotype model proposed by Fried and coworkers which consists of a five‐item questionnaire whereby the patients are classified as robust, pre‐frail or frail if they fulfill none, one or two, and more than two criteria, respectively.^19^

The five criteria considered were: presence of fatigue evaluated by self‐report questions of the Center for Epidemiological Studies Depression; unintentional loss of weight (≥4.5 kg or ≥5% of total weight in the preceding year); low physical activity defined by the International Physical Activity Questionnaire (IPAQ) as less than ten consecutive minutes of any activity in the last week; slow gait, measured by walking time in seconds (a distance of 4.6 m adjusted for sex and height); and reduced grip strength measured with a dynamometer (Jamar Hydraulic Hand Dynamometer) in the dominant hand. Reference values were extracted from a local population study and adjusted for sex, height, and body mass index (BMI).^20^

### 2.3 Blood pressure assessment

Ambulatory blood pressure monitoring was conducted using an automated and validated device (Spacelabs Medical, model 90207) which was adapted to record BP every 15 minutes during the day and every 30 minutes during the night. A cuff of appropriate size was installed on the non‐dominant arm according to current guidelines.^1^

The examination was considered appropriate if at least 75% of the systolic and diastolic readings were successful with at least 16 valid readings while awake and eight valid readings while asleep according to local guidelines.^21^ The mean of all valid readings was used for analysis. Awake and sleep periods were defined according to times reported in the patient´s diary.

Patients were considered hypertensive when they used any antihypertensive medication or in the presence of systolic BP ≥140 mm Hg or diastolic ≥90 mm Hg on two different occasions.^1^ Asleep systolic BP fall was measured as percentage [(awake BP – asleep BP)/awake BP]. Nocturnal dipping was considered present if systolic BP fall was ≥10%.^21^

### 2.4 Statistical analysis

Continuous data were tested for normality by the Shapiro Wilk test and reported as mean ± standard deviation or as median (interquartile range) accordingly. Qualitative values are reported as absolute frequency and percentages. Continuous variables were compared between groups with Student t test or Mann‐Whitney according to normality. Pearson Chi‐Square test was applied for categorical variables comparison between groups.

Multiple unadjusted logistic regression tests were performed by logistic regression and linear models considering non‐dipping and systolic BP fall as dependent variables. A first model was created for adjustment with all the clinically relevant variables. In accordance with the number of individuals evaluated, which limits the number of covariates that can be added to logistic regression models, a second model was created with lesser covariates of adjustment with only the clinically relevant variables or with the variables that showed positive correlation in an unadjusted analysis. Coefficient was used to estimate the effect of each variable over the dependent variable. The covariables considered for adjusted analysis were: presence of frailty, age, body mass index (BMI), number of antihypertensive medications, presence of hypertension, 24‐hour systolic BP and orthostatic hypotension.

The analyses were performed using the SAS 9.4 (SAS Institute, Cary, NC, USA) and SPSS 25 (IBM Corp., Armonk, NY, USA) software, with the level of significance set at *p* < .05.

## 3 RESULTS

Mean age was 85.3 ± 3,7 years; 38 patients (51.4%) were found to be frail and 36 (48.6%) were robust. The female sex was predominant, accounting for 50 (67.5%) individuals. Fifty‐four (72,9%) were of white ethnicity, 58 (78,3%) were hypertensive, and 21 (28,3%) had type 2 diabetes. There was homogeneity for most of the demographic characteristics such as age, BMI, abdominal circumference, diabetes, and number of antihypertensives taken among frail and robust patients. However, the median number of comorbidities was seven (5–9) for the frail group compared to four (3–6) (*p* < .01) for the robust group, with 89% and 67% of hypertensive patients (*p* = .02), respectively. The demographic characteristics and distribution between groups are summarized in Table 1.

**TABLE 1:** Characteristics of study population

	Frailty			
Variable	No (*n* = 36)	Yes (*n* = 38)	*p*‐value	Total (*n* = 74)
Age (years)	84.78 ± 3.39	85.84 ± 4.02	.22	85.32 ± 3.74
*Sex*				
Female	21 (58.3%)	29 (76.3%)	.09	50 (67.6%)
*Ethnicity*			.8	
White	27 (75%)	27 (71%)		54 (72.9%)
Black	3 (8.3%)	5 (13.1%)		8 (10.8%)
BMI (Kg/m^2^)	27 ± 4,3	27 ± 5,12	.76	27 ± 4.71
Abdominal circumference (cm)	98 ± 12	101 ± 12	.27	99 ± 12
Num. comorbidities	4 (3 ‐ 6)	7 (5 ‐ 9)	<.01	5 (4 ‐ 7)
Num. antihypertensives	2 (0 ‐ 2)	1 (1 ‐ 2)	.24	1 (1 ‐ 2)
Hypertension	24 (66.6%)	34 (89.5%)	.02	58 (78.3%)
Diabetes	8 (22.2%)	13 (34.2%)	.25	21 (28.3%)
*Office blood pressure*				
Sitting SBP (mm Hg)	142 ± 22	135 ± 17	.11	138 ± 20
Sitting DBP (mm Hg)	73 ± 11	69 ± 12	.09	71 ± 12
Laying SBP (mm Hg)	143 ± 24	136 ± 17	.13	140 ± 21
Laying DBP (mm Hg)	74 ± 12	69 ± 11	.03	71 ± 12
Standing SBP (mm Hg)	145 ± 23	137 ± 21	.13	141 ±22
Standing DBP (mm Hg)	78 ± 13	71 ± 12	.02	75 ± 13
Orthostatic Hypotension	7 (19%)	8 (21%)	.86	15 (20%)

Data are presented as mean ± SD or median (IQR) for continuous variables, or n (%) for categorical variables.

*p* < .05 between frail and non‐frail individuals.

*Abbreviations*: BMI, body mass index; DBP, diastolic blood pressure; Num., number of.; SBP, systolic blood pressure.

Analysis of office measurements showed no difference between groups in mean systolic BP in the sitting, lying, or standing position neither in the prevalence of orthostatic hypotension. The frail population had lower diastolic pressure in the lying (69 ± 11 mm Hg vs. 74 ± 12 mm Hg P = 0,03) and standing (71 ± 12 mm Hg vs. 78 ± 13 mm Hg; P = 0,02) positions (Table 1).

Twenty‐four‐hour readings of systolic and diastolic BP, mean arterial pressure, pulse pressure and heart rate were similar between the two groups. Awake systolic and diastolic BP were similar for frail and robust individuals. In contrast, mean systolic BP during sleep was higher among frail patients (128 ± 15 mm Hg vs. 122 ±13 mm Hg *p* = .04), whereas no difference was detected for asleep diastolic BP. Frailty was associated with a greater prevalence of non‐dipping status [36 (61%) vs. 23 (39%) *p* < .01] and reduced systolic BP fall when compared to the robust population [1 (‐4.5 – 5)% vs. 6.8 (2.1 – 12.8)% *p* = < .01]. The ABPM findings are summarized in Table 2.

**TABLE 2:** Ambulatory blood pressure monitoring findings

	Frailty			
Variable	No (*n* = 36)	Yes (*n* = 38)	*p*‐value	Total
24h SBP (mm Hg)	129 ± 11	128 ± 13	.85	128 ± 12
24h DBP (mm Hg)	70 ± 8	68 ± 8	.23	69 ± 8
24h MAP (mm Hg)	91 ± 8	90 ± 8	.33	91 ± 8
24h PP (mm Hg)	59 ± 10	60 ± 11	.54	60 ± 10
24h HR (bpm)	74 ± 9	74 ± 11	.89	74 ± 10
Awake SBP (mm Hg)	131 ± 11	128 ± 13	.34	130 ± 12
Awake DBP (mm Hg)	72 ± 8	68 ± 8	.51	70 ± 8
Asleep SBP (mm Hg)	122 ± 13	128 ± 15	.04	125 ± 14
Asleep DBP (mm Hg)	64 ± 10	66 ± 9	.28	65 ± 9
SBP Nondipping	23 (39%)	36 (61%)	<.01	59 (79%)
SBP nocturnal decrease, %	6.8 (2.1 – 12.8)	1 (‐4.5 – 5)	<.01	3.4 (‐0.7 – 8.7)

Data are mean ± SD or median (interquartile range) for continuous variables or n (%) for categorical variables.

*p* < .05 between frail and non‐frail individuals.

*Abbreviations*: BPM, beats per minute.; DBP, diastolic blood pressure; HR, heart rate; MAP, mean arterial pressure; PP, pulse pressure; SBP, systolic blood pressure.

Clinically relevant variables were analyzed by logistic regression to find association with the risk of non‐dipping. Frailty (OR 10.2; CI 2.1 – 49.29; *p* < .01) and BMI (OR 1.1; CI 1.04 – 1.32; *p* < .01) were associated with a higher risk in an unadjusted analysis. In a multivariate model, frailty (OR 12.4; CI 1.79 – 85.9; *p* = .01) and BMI (OR 1.2; CI 1.28 – 1.6; *p* = .02) were independently associated with a higher risk of non‐dipping even after adjustment for age, presence of hypertension, number of antihypertensives taken, 24‐hour systolic BP and presence of orthostatic hypotension. In accordance with the number of individuals analyzed, a second model was created with only the presence of frailty and BMI to avoid overfeeding. The second model showed persistent association of the risk for either frailty (OR 13.7; CI 2.24 – 83.83; *p* < .01) and BMI (OR 1.2; CI 1.05 – 1.43; *p* < .01) with stronger association of the former. All these analyzes are shown in Table 3.

**TABLE 3:** Logistic regression models for the risk of nondipping

	Not adjusted			Model 1			Model 2		
	OR	CI	*p*‐value	OR	CI	*p*‐value	OR	CI	*p*‐value
Frailty	10.2	2.1 – 49.29	<.01	12.4	1.79 – 85.9	.01	13.7	2.24 – 83.83	<.01
Age, years	1	0.87 – 1.14	.85	1	0.86 – 1.33	.53	–	–	–
BMI, Kg/m^2^	1.1	1.04 – 1.32	<.01	1.2	1.28 –1.6	.02	1.2	1.05 – 1.43	<.01
Hypertension	3.1	0.9 – 10.81	.07	5.4	0.33 – 87.4	.23	–	–	–
Num. antihypertensives	1.2	0.65 – 2.37	.5	0.5	0.16 – 1.8	.32	–	–	–
24h–SBP (mm Hg)	1	0.95 – 1.05	.9	0.9	0.9 – 1.04	.41	–	–	–
Orthostatic hypotension	1.8	0.36 – 9.19	.45	2.3	0.38 – 14.7	.39	–	–	–

*p* < .05 indicates higher risk for nondipping.

Covariables included for adjustment in model 1: frailty, age, BMI, hypertension, num. antihypertensives, 24h‐SBP, orthostatic hypotension.

Covariables included for adjustment in model 2: frailty and BMI.

*Abbreviations*: BMI, body mass index; CI, confidence interval; OR, odds ratio; SBP, systolic blood pressure.

The effect of each variable over systolic BP fall was individually estimated by coefficient in logistic regression as shown in Table 4. The presence of frailty was estimated to reduce BP fall by ‐7.2% (CI ‐10.1 – ‐3.6 % *p* < .01) whereas the presence of hypertension by ‐5.7% (CI ‐10 – ‐1.2% *p* = .01). In a multivariate model, frailty was independently associated with a reduce in BP fall by ‐6.1% (CI ‐9.6 – ‐2.6 % *p* = .01) even after adjustment for age, BMI, presence of hypertension, number of antihypertensives, 24h systolic BP and orthostatic hypotension. The effect was maintained and strengthen in a second model with lesser variables: ‐6.4% (CI ‐10 – ‐2.8% *p* < .01).

**TABLE 4:** Estimated effect on systolic blood pressure decrease during sleep

	Not Adjusted			Model 1			Model 2		
Variable	Effect(%)	CI	*p*	Effect(%)	CI	*p*	Effect(%)	CI	*p*
Frailty^a^	−7.2	−10.1 – −3.6	<.01	−6.1	−9.6 – −2.6	.01	−6.4	−10 – −2.8	<.01
Age^b^	−0.4	−0.9 – 0.1	.1	−0.4	−0.9 – 0.1	.12	–	–	–
BMI^b^	−0.4	−0.7 – 0.1	.18	−0.3	−0.7 – 0.0	.06	–	–	–
Hypertension^a^	−5.7	−10 – −1.2	.01	0.1	−7 – 7.3	.97	−3.5	−7.6 – 0.6	.09
Antihypertensives^b^	−2.4	−5 – 0.2	.06	−1.8	−5.4 – 1.7	.31	–	–	–
24h SBP (mm Hg)^b^	0	−0.2 – 0.2	.87	0	−0.1 – 0.2	.84	–	–	–
Ort. Hypotension^a^	−1	−6.4 – 4.4	.7	−0.5	−5.2 – 4.2	.84	–	–	–

*p* < .05 indicates valid effect of the variable over systolic BP decrease.

Covariables included for adjustment in model 1: frailty, age, BMI, hypertension, num. antihypertensives, 24h‐SBP, orthostatic hypotension.

Covariables included for adjustment in model 2: frailty and hypertension.

*Abbreviations*: BMI, body mass index; CI, confidence interval; Ort. Hypotension, orthostatic hypotension.

^a^ Change in blood pressure decrease for the presence of the categorical variable.

^b^ Change in blood pressure decrease per 1 unit change in the continuous variable.

Separate sensitivity analyses were performed with linear regressions considering the effect of awake systolic BP in non‐dipping status and systolic BP fall but no association was found.

## 4 DISCUSSION

This was a cross‐sectional study, designed to determine the role of frailty on the 24‐hour blood pressure pattern in a population above 80 years old, with specific focus on circadian variations. The results showed no difference in office measures for systolic BP but substantial influence of frailty on elevated asleep systolic BP and its lack of decrease in the very elderly. These data support the evidence of frailty as an important instrument for assessing cardiovascular risk in this heterogenous population and can be used in clinical practice, and further clinical trials, to guide treatment decisions. It also emphasizes the importance of sleep on future investigations and treatment of frailty and hypertension.

The groups were homogenous in terms of demographic characteristics such as frailty, age, sex, BMI, and abdominal circumference, which allowed reducing biases. Despite a predominance of hypertension in the frail population (89.5% vs. 66.6%, *p* = .02), previous studies show that frailty is associated with hypertension.^6^, ^22^ The number of comorbidities per individual was found to be greater in the frail population [7 (5–9) vs. 4 (3–6), *p* < .01] which might be explained by the strong association between comorbidities and frailty as proposed by Rockwood and coworkers^23^

Office measurements revealed no difference between groups in systolic BP measured in different positions neither in the prevalence of orthostatic hypotension. Similar results were obtained in a cross‐sectional study which analyzed office BP in 200 individuals stratified by three different frailty instruments.^24^ Our study complements the evidence that considering office BP as a single parameter for cardiovascular risk in individuals above 80 years could underestimate the true risk in this population by disregarding the circadian cycle which plays an important role in the physiopathology between frailty and cardiovascular diseases. The reduced diastolic BP in the frail group could be explained by the effect of antihypertensives on a more resistant and less compliant endothelial system, as seen in cardiovascular aging.^25^

The ABPM findings in this study showed that frailty was associated with higher asleep systolic BP and a higher risk of non‐dipping even after adjustment for confounding variables. Recent evidence suggests that elevated asleep systolic BP and the lack of BP fall are mainly influenced by diurnal physical activity and changes in sleep patterns.^26^, ^27^, ^28^ Considering that frailty also exerts important effect on sleep and mobility, this conjunction might be a plausible explanation for these findings. The higher risk associated with BMI also suggests that sleep disorders, like undiagnosed obstructive sleep apnea, might be associated with these findings since it has strong association with overweight.^29^ Despite no difference in the prevalence of diabetes or orthostatic hypotension between groups in our findings, conditions associated with blood pressure non‐dipping pattern, other plausible explanations would be advanced endothelial dysfunction associated with impairment in the autonomic system and insulin resistance regularly found in the frail.^30^ The overall presence of dipping was low when compared to a general population and might be explained, in addition to what was previously mentioned, by the effect of age, since older individuals tend to have less dipping.^31^

The strong and independent effect of frailty on BP fall supports that even with these possible mechanisms involved, frailty plays an independent role over cardiovascular disease and must be considered as a crucial aspect when evaluating older adults, especially the very elderly. The American Heart Association raises the issue by pointing to the heterogeneity of this population and by suggesting emphasis in studies that include the role of frailty in cardiovascular diseases.^32^ Since they have similar risk factors there is a bi‐directional relation between them that supports this perspective, furthermore, frailty has been proved to predict mortality and hospitalization for cardiovascular diseases.^33^, ^34^ The estimated effect of frailty over nocturnal BP decrease in our findings, ‐6.1% (CI ‐9.6 – ‐2.6 % *p* = .01), might represent a 20% higher risk of cardiovascular mortality in 9 years according to previous prospective study.^35^

To our knowledge, this was the first study to analyze the role of frailty on the 24‐hour blood pressure pattern in the very elderly population. The relatively small number of patients evaluated could be cited as one of the major limitations of the present study. However, there was great difficulty of finding very elderly individuals with the profile of this research and who agreed to participate in it. The exclusion of pre‐frail patients hinders extrapolation to a general population of the same age, in contrast, it emphasizes the effect of frailty on the dependent variables. The cross‐sectional design did not permit establishing a causal relationship, neither it allowed control of medications, a fact that could hinder interpretation of the data, although statistical analysis was used to adjust for possible confounding factors. The higher prevalence of hypertension in the frail could be cited as possible bias; however, this was compatible with previous studies in a similar population and after statistical adjustments with frailty and other variables, the effect of hypertension over the dependent variables did not prevail. Since comorbidities are implied in some definitions of frailty, it was not possible to equalize nor adjust this variable between groups.

Further studies objectively measuring daily mobility and sleep quality in this specific population are suggested for a better understanding of this patient. Based in the present study, a focus in frailty and sleep treatment could be cited as promising adjuvant therapies for cardiovascular disease in the very elderly, although intervention trials designed to include frailty are warranted to clarify treatment.

## 5 CONCLUSIONS

In conclusions, frailty has a substantial influence on nighttime BP values and pattern in patients older than 80 years.

## CONFLICT OF INTEREST

The authors declare no conflict of interest.
